# Using SVD on Clusters to Improve Precision of Interdocument Similarity Measure

**DOI:** 10.1155/2016/1096271

**Published:** 2016-08-07

**Authors:** Wen Zhang, Fan Xiao, Bin Li, Siguang Zhang

**Affiliations:** ^1^Center on Big Data Sciences, Beijing University of Chemical Technology, Beijing 100039, China; ^2^Institute of Policy and Management, Chinese Academy of Sciences, Beijing 100190, China

## Abstract

Recently, LSI (Latent Semantic Indexing) based on SVD (Singular Value Decomposition) is proposed to overcome the problems of polysemy and homonym in traditional lexical matching. However, it is usually criticized as with low discriminative power for representing documents although it has been validated as with good representative quality. In this paper, SVD on clusters is proposed to improve the discriminative power of LSI. The contribution of this paper is three manifolds. Firstly, we make a survey of existing linear algebra methods for LSI, including both SVD based methods and non-SVD based methods. Secondly, we propose SVD on clusters for LSI and theoretically explain that dimension expansion of document vectors and dimension projection using SVD are the two manipulations involved in SVD on clusters. Moreover, we develop updating processes to fold in new documents and terms in a decomposed matrix by SVD on clusters. Thirdly, two corpora, a Chinese corpus and an English corpus, are used to evaluate the performances of the proposed methods. Experiments demonstrate that, to some extent, SVD on clusters can improve the precision of interdocument similarity measure in comparison with other SVD based LSI methods.

## 1. Introduction

As computer networks become the backbones of science and economy, enormous quantities of machine readable documents become available. The fact that about 80 percent of businesses are conducted on unstructured information [1, 2] makes the great demand for the efficient and effective text mining techniques which aims to discover high quality knowledge from unstructured information. Unfortunately, the usual logic-based programming paradigm has great difficulties in capturing fuzzy and often ambiguous relations in text documents. For this reason, text mining, which is also known as knowledge discovery from texts, is proposed to deal with uncertainness and fuzziness of languages and disclose hidden patterns (knowledge) in documents.

Typically, information is retrieved by literally matching terms in documents with those of a query. However, lexical matching methods can be inaccurate when they are used to match a user's query. Since there are usually many ways to express a given concept (synonymy), the literal terms in a user's query may not match those of a relevant document. In addition, most words have multiple meanings (polysemy and homonym), so terms in a user's query will literally match terms in irrelevant documents. For these reasons, a better approach would allow users to retrieve information on the basis of a conceptual topic or meanings of a document [3, 4].

Latent Semantic Indexing (LSI) is proposed to overcome the problem of lexical matching by using statistically derived conceptual indices instead of individual words for retrieval [5, 6]. We call this retrieval method Latent Semantic Indexing because the subspace represents important associative relationships between terms and documents that are not evident in individual documents. LSI assumes that there is some underlying or latent structure in word usage that is partially obscured by variability in word choice. Using the singular value decomposition (SVD), one can take advantage of the implicit higher-order structure in the association of terms with documents by determining the SVD of large sparse term-document matrix. Terms and documents represented by a reduced dimension of the largest singular vectors are then matched against user queries. Performance data shows that the statistically derived term-document matrix by SVD is more robust to retrieve documents based on concepts and meanings than the original term-document matrix produced using merely individual words with vector space model (VSM).

In this paper, we propose SVD on clusters (SVDC) to improve the discriminative power of LSI. The contribution of this paper is three manifolds. Firstly, we make a survey of existing linear algebra methods for LSI, including both SVD based methods and non-SVD based methods. Secondly, we theoretically explain that dimension expansion of document vectors and dimension projection using SVD are the two manipulations involved in SVD on clusters. We develop updating processes to fold in new documents and terms in a decomposed matrix by SVD on clusters. Thirdly, two corpora, a Chinese corpus and an English corpus, are used to evaluate the performances of the proposed methods.

The rest of this paper is organized as follows.  Section 2 provides a survey on recent researches on Latent Semantic Indexing and its related topics.  Section 3 proposes SVD on clusters and its updating process.  Section 4 is the experiment to evaluate the proposed methods.  Section 5 concludes this paper and indicates future work.

## 2. Related Work

### 2.1. Singular Value Decomposition

The singular value decomposition is commonly used in the solution of unconstrained linear least square problems, matrix rank estimation, and canonical correlation analysis [7, 8]. Given *m* × *n* matrix *A*, where without loss of generality *m* ≥ *n* and rank(*A*) = *r*, the singular value decomposition of *A*, denoted by SVD(*A*), is defined as

(1) $$\begin{array}{c} A=U\Sigma V^{T}. \end{array}$$

Here *U* ^*T*^ *U* = *V* ^*T*^ *V* = *I*_*n*_ and Σ = diag⁡(*σ*_1_,…, *σ*_*n*_), *σ*_*i*_ > 0 for 1 ≤ *i* ≤ *r* and *σ*_*j*_ > 0 for *j* ≥ *r* + 1. The first *r* columns of the orthonormal matrices *U* and *V* define the orthonormal eigenvector associated with *r* nonzero eigenvalues of *AA* ^*T*^ and *A* ^*T*^ *A*, respectively. The columns of *U* and *V* are referred to as the left and right singular vectors, respectively, and the singular values of *A* are defined as the diagonal elements of Σ which are the nonnegative square roots of the *n* eigenvalues of *AA* ^*T*^. Furthermore, if we define *A*_*k*_ = ∑_*i*=1_^*k*^ *u*_*i*_ *δ*_*i*_ *v*_*i*_^*T*^, then we will find that *A*_*k*_ is the best rank-*k* approximation for *A* in terms of Frobenius norm [7].

### 2.2. Recent Studies in LSI

Recently, a series of methods based on different methods of matrix decomposition have been proposed to conduct LSI. A common point of these decomposition methods is to find a rank-deficient matrix in the decomposed space to approximate the original matrix so that the term frequency distortion in term-document can be adjusted. Basically, we can divide these methods into two categories: matrix decomposition based on SVD and matrix decomposition not based on SVD.  Table 1 lists the existing linear algebraic methods for LSI.

In the aspect of SVD based LSI methods, it includes IRR [9], SVR [10], and ADE [11]. Briefly, IRR conjectures that SVD removes two kinds of “noises” from the original term-document matrix: exceptional documents and documents with minor terms. However, if our concentration is on characterizing relationships of documents in a collection rather than looking for representative documents, then IRR can play an effective role for this work. The basic idea behind SVR is that the “noise” in original document representation vectors comes from minor vectors, that is, those vectors which are far from representative vectors in terms of distance. Thus, we need to augment the influence of representative vectors and meanwhile reduce the influence of minor vectors in the approximation matrix. Following this idea, SVR adjusts the differences among major dimensions and minor dimensions in the approximation matrix by rescaling the singular values in Σ. Based on the observation that singular values in Σ have the characteristics as low-rank-plus-shift structure, ADE tries to flatten the first *k* largest singular values with a fixed value and combine with other small singular values to reconstruct Σ to make dimension values relatively equalized in the approximation matrix of *A*.

In the aspect of non-SVD based LSI methods, it includes SDD [12], LPI [13], and R-SVD [14]. SDD restricts values in singular vectors (*U* and *V*) in approximation matrix only having entries in the set {−1, 0, 1}. By this way, it merely needs one-twentieth of storage and only one-half query time while it can do and SVD does LSI in terms of information retrieval. LPI argues that LSI seeks to uncover the most representative features rather the most discriminative features for document representation. With this motivation, LPI constructs the adjacency graph of documents and aims to discover the local structure of document space using Local Preserving Projection (LPP). In essence, we can regard that LPI is adapted from LDA (Linear Discriminant Analysis) [15], which is a topic concerning dimension reduction for supervised classification. R-SVD is different with SVD mathematically in that the term-document matrix decomposition of SVD is based on Total Least Square (TLS) while matrix decomposition in R-SVD is based on Structured Total Least Square (STLS). R-SVD is not designed for LSI but for information filtering to improve the effectiveness of information retrieval by using users' feedback.

Recently, two methods in [16, 17] are presented which also make use of SVD and clustering. In [16], Gao and Zhang investigate three strategies of using clustering and SVD for information retrieval as noncluster retrieval, full-cluster retrieval, and partial cluster retrieval. Their study shows that partial cluster retrieval produces the best performance. In [17], Castelli et al. make use of clustering and singular value decomposition for nearest-neighbor search in image indexing. They use SVD to rotate the original vectors of images to produce zero-mean, uncorrelated features. Moreover, a recursive clustering and SVD strategy is also adopted in their method when the distance of reconstructed centroids and original centroids exceeds a threshold.

Although the two methods are very similar with SVD on clusters, they are proposed for different uses with different motivations. Firstly, this research presents a complete theory for SVD on clusters, including theoretical motivation, theoretical analysis of effectiveness, and updating process, which are entirely not mentioned in any of the two referred methods. Secondly, this research describes the detailed procedures of using SVD on clusters and attempts to use different clustering methods (*k*-Means and SOMs clustering), which are not mentioned in any of the two referred methods, either. Thirdly, the motivations of proposing SVDC are different with theirs. They proposed clustering and SVD for inhomogeneous data sets and our motivation is to improve the discriminative power of document indexing.

## 3. SVD on Clusters

### 3.1. The Motivation

The motivation for the proposal of SVD on clusters can be specified as the following 4 aspects:

(1) The huge computation complexity involved in traditional SVD. According to [18], the actual computation complexity of SVD is quadratic in the rank of term-document matrix (the rank is bounded by the smaller of the number of documents and the number of terms) and cubic in the number of singular values that are computed [19]. On the one hand, in most cases of SVD for a term-document matrix, the number of documents is quite smaller than the number of index terms. On the other hand, the number of singular values, which is equal to the rank of the term-document matrix, is also dependent on the number of documents. For this reason, we can regard that the computation complexity of SVD is completely determined by the number of documents in the term-document matrix. That is to say, if the number of documents in the term-document matrix is reduced, then the huge computation complexity of SVD can be reduced as well.
(2) Clusters existing in a document collection. Usually, there are different topics scattered in different documents of a text collection. Even if all documents in a collection are concerning on a same topic, we can divide them into several subtopics. Although SVD has the ability to uncover the most representative vectors for text representation, it might not be optimal in discriminating documents with different semantics. In information retrieval, the relevant documents with the query should be retrieved as many as possible; on the other hand, the irrelevant documents with the query should be retrieved as few as possible. If principal clusters, in which documents have closely related semantics, can be extracted automatically, then relevant documents can be retrieved in the cluster with the assumption that closely associated documents tend to be relevant to the same request; that is, relevant documents are more like one another than they are like nonrelevant documents.
(3) Contextual information and cooccurrence of index terms in documents. Classic weighting schemes [20, 21] are proposed on the basis of information about the frequency distribution of index terms within the whole collection or within the relevant and nonrelevant sets of documents. The underlying model for these term weighting schemes is a probabilistic one and it assumes that the index terms used for representation are distributed independently in documents. Assuming variables to be independent is usually a matter of mathematical convenience. However, in the nature of information retrieval, exploitation of dependence or association between index terms or documents will often lead to a better retrieval results such as most linear algebra methods proposed for LSI [3, 22]. That is, from mathematical point of view, the index terms in documents are dependent on each other. In the viewpoint of linguistics, topical words are prone to have burstiness in documents and lexical words concerning the same topic are likely to cooccur in the same content. That is, the contextual words of an index term should also be emphasized and put together when used for retrieval. In this sense, capturing the cooccurrence of index terms in documents and further capturing the cooccurrence of documents with some common index terms are of great importance to characterize the relationships of documents in a text collection.
(4) Divide-and-conquer strategy as theoretical support. The singular values in Σ of SVD of term-document matrix *A* have the characteristic as low-rank-plus-shift structure; that is, the singular values decrease sharply at first, level off noticeably, and dip abruptly at the end. According to Zha et al. [23], we know that if *A* has the low-rank-plus-shift structure, then the optimal low-rank approximation of *A* can be computed via a divide-and-conquer approach. That is to say, approximation of submatrices of *A* can also produce comparable effectiveness in LSI to direct SVD of *A*.

With all of the above observations from both practices and theoretical analysis, SVD on clusters is proposed for LSI to improve its discriminative power in this paper.

### 3.2. The Algorithms

To proceed, the basic concepts adopted in SVD on clusters are defined in the following in order to make clear the remainder of this paper.

**Definition 1 (cluster submatrix).**

Assuming that *A* is a term-document matrix, that is, *A* = (*d*_1_, *d*_2_,…, *d*_*n*_) (*d*_*i*_  (1 ≤ *i* ≤ *n*) is a term-document vector), after clustering process, *n* document vectors are partitioned into *k* disjoint groups (each document belongs to only one group but all the documents have the same terms for representation). For each of these clusters, a submatrix of *A* can be constructed by grouping the vectors of documents which are partitioned into the same cluster by clustering algorithm. That is, *A* = [*A* ^(1)^, *A* ^(2)^,…, *A* ^(*k*)^], due to the fact that changing the order of documents vectors in *A* can be ignored. Then, one calls that *A* ^(*j*)^  (1 ≤ *j* ≤ *k*) is a cluster submatrix of *A*.

**Definition 2 (SVDC approximation matrix).**

Assuming that *A* ^(1)^, *A* ^(2)^,…, *A* ^(*k*)^ are the all cluster submatrices of *A*, that is, *A* = [*A* ^(1)^, *A* ^(2)^,…, *A* ^(*k*)^], after SVD for each of these cluster submatrices, that is, *A* ^(1)^ ≈ *A*_*r*_1__^(1)^, *A* ^(2)^ ≈ *A*_*r*_2__^(2)^,…, *A* ^(*k*)^ ≈ *A*_*r*_*k*__^(*k*)^, and *r*_*k*_ is the rank of SVD approximation matrix of *A* ^(*k*)^ and, *A*_*r*_*k*__^(*k*)^ is the SVD approximation matrix of *A* ^(*k*)^, then one calls that $\bar{A}=[A_{r_{1}}^{\left(1\right)},A_{r_{2}}^{\left(2\right)},\ldots ,A_{r_{k}}^{\left(k\right)}]$ is a SVDC approximation matrix of *A*.

With the above two definitions of cluster submatrix and SVDC approximation matrix, we proposed two versions of SVD on clusters by using *k*-Means clustering [24] and SOMs (Self-Organizing Maps) clustering [25]. These two versions are illustrated in Algorithms 3 and 4, respectively. The difference of these two versions lies in different clustering algorithms used in them. For *k*-Means clustering, we need to predefine the number of clusters in the document collection and for SOMs clustering, it is not necessary to predefine the number of clusters beforehand.

**Algorithm 3 .**

Algorithm of SVD on clusters with *k*-Means clustering to approximate the term-document matrix for LSI is as follows:

 Input:
   
*A* is term-document matrix; that is, *A* = (*d*_1_, *d*_2_,…, *d*_*n*_).
   
*k* is predefined number of clusters in *A*.
   
*r*_1_, *r*_2_,…, *r*_*k*_ are predefined rank of SVD approximation matrix for *k* clusters submatrices of *A*.
 Output:
   
$\overset{-}{A}$ is the SVDC approximation matrix of *A*.
 Method:
  (1) Cluster the document vectors *d*_1_, *d*_2_,…, *d*_*n*_ into *k* clusters using *k*-Means clustering algorithm.
  (2) Allocate the document vectors according to vectors' cluster labels from *A* to construct the cluster submatrices (*A* ^(1)^, *A* ^(2)^,…, *A* ^(*k*)^).
  (3)  Conduct SVD for each of the cluster submatrices of *A* ^(*i*)^  (1 ≤ *i* ≤ *k*) and produce their SVD approximation matrix, respectively. That is, *A* ^(*i*)^ ≈ *A*_*r*_*i*__^(*i*)^.
  (4)  Merge all the SVD approximation matrices of the cluster submatrices to construct the SVDC approximation matrix of *A*. That is, $\overset{-}{A}=\left[A_{r_{1}}^{\left(1\right)},A_{r_{2}}^{\left(2\right)},\ldots ,A_{r_{k}}^{\left(k\right)}\right]$.

### 3.3. Theoretical Analysis of SVD on Clusters

For simplicity, here, we only consider the case that term-document *A* is clustered into two cluster submatrices *A*_1_ and *A*_2_; that is, *A* = [*A*_1_, *A*_2_]. After SVD processing for *A*_1_ and *A*_2_, we obtain *A*_1_ = *U*_1_Σ_1_ *V*_1_^*T*^ and *A*_2_ = *U*_2_Σ_2_ *V*_2_^*T*^. For convenience of explanation, if we assume that

(2) A′=A100A2,U′=U100U2Σ′=Σ100Σ2V′T=V1′T00V2′T,

we will obtain that *A*′ = *U*′Σ′*V* ^′*T*^ and *U* ^′*T*^ *U*′ = *V* ^′*T*^ *V*′ = *I*_*n*_; that is, *U*′ and *V*′ are orthogonal matrices. Hence, we will also obtain

(3) $$\begin{array}{c} A^{\prime }=\overset{r^{\prime }}{\underset{i=1}{\sum }}\sigma_{i}^{\prime }u_{i}^{\prime }v_{i}^{\prime T}, \end{array}$$

where *r*′ is the total number of elements in Σ_1_ and Σ_2_ which are nonzeros. Thus, we can say that *A*′ = *U*′Σ′*V* ^′*T*^ is a singular decomposition of *A*′ and *A*_*k*_′ = ∑_*i*=1_^*k*^ *σ*_*i*_′*u*_*i*_′*v*_*i*_^′*T*^ is the closet rank-*k* approximation for *A*′ in terms of Frobenius norm (assuming that we sort the values in Σ′ in descending order and adapt the orders of *u*_*i*_′ and *v*_*i*_′ accordingly).

We can conclude that there are actually two kinds of manipulations involved in SVD on clusters: the first one is dimension expansion of document vectors and the second one is dimension projection using SVD.

On the one hand, notice that *A* ∈ *R* ^*m*×*n*^ and *A*′ ∈ *R* ^2*m*×*n*^, *A*′ has expanded *A* into another space where the number of dimensions is twice as that of the original space of *A*. That is, in *A*′, we expanded each document vector *d* into *R* ^2*m*^ dimension vector *d*′ by

(4) $$\begin{array}{c} d_{p}^{\prime }=\left\{\begin{array}{cc} d_{q}, & \text{if }d\in C_{i},\;p=\left(i-1\right)m+q\\ 0, & \text{otherwise}. \end{array}\right. \end{array}$$

Here, *d*_*q*_ is the value of *q*th dimension in *d*, *d*_*p*_′ is the value of *p*th dimension of *d*′, and 1 ≤ *i* ≤ 2. In this way, we expanded each *d* into *R* ^2*m*^ dimension vector *d*′ where values of *d*′ are equal to the corresponding values of *d*, if *d* belongs to cluster *C*_*i*_ or zero, if *d* is not a member of that cluster.

Theoretically, according to the explanation, document vectors which are not in the same cluster submatrix will have zero cosine similarity. However, in fact, all document vectors have the same terms in representation and dimension expansion of document vectors is derived by merely copying the original pace of *A*. For this reason, in practice, we use the vectors in *A*_1_ and *A*_2_ for indexing and cosine similarities of document vectors in *A*_1_ and *A*_2_ will not necessarily be zero. This validates our motivation of using similarity measure for LSI performance evaluation in Section 4.2.

**Algorithm 4 .**

Algorithm of SVD on clusters with SOMs clustering to approximate the term-document matrix for LSI is as follows:

 Input:
   
*A* is term-document matrix; that is, *A* = (*d*_1_, *d*_2_,…, *d*_*n*_).
   
*α* is predefined preservation rate for submatrices of *A*.
 Output:
   
$\overset{-}{A}$ is the SVDC approximation matrix of *A*.
 Method:
  (1) Cluster the document vectors *d*_1_, *d*_2_,…, *d*_*n*_ into clusters using SOMs clustering algorithm.
  (2) Allocate the document vectors' according to vectors' cluster labels from *A* to construct the cluster submatrices (*A* ^(1)^, *A* ^(2)^,…, *A* ^(*k*)^) (notice here that *k* is not a predefined number of clusters of *A* but the number of neurons which are matched with at least 1 document vector).
  (3)  Conduct SVD using predefined preservation rate for each cluster submatrix of *A* ^(*i*)^  (1 ≤ *i* ≤ *k*) and produce its SVD approximation matrix. That is, *A* ^(*i*)^ ≈ *A*_*α*_^(*i*)^.
  (4)  Merge all the SVD approximation matrices of the cluster submatrices to construct the SVDC approximation matrix of *A*. That is, $\overset{-}{A}=\left[A_{\alpha }^{\left(1\right)},A_{\alpha }^{\left(2\right)},\ldots ,A_{\alpha }^{\left(k\right)}\right]$.

On the other hand, when using SVD for *A*, that is, *A* = *U*Σ*V* ^*T*^, we obtain *U* ^*T*^ *A* = Σ*V* ^*T*^ and further we say that SVD has folded each document vector of *A* into a reduced space (assuming that we use *U*_*k*_^*T*^ for the left multiplication of *A*, the number of dimensions of original document vectors will be reduced to *k*), which is represented by *U* and reflects the latent semantic dimensions characterized by term cooccurrence of *A* [3]. In the same way, for *A*′, we have *U* ^′*T*^ *A* ^′*T*^ = Σ*V* ^′*T*^ and further we may say that *A*′ is projected into space which is represented by *U*′. However, here *U*′ is not characterized by term cooccurrence of *A*′ but by the existing clusters of *A* and the term cooccurrence of each cluster submatrix of *A*.

### 3.4. The Computation Complexity of SVD on Clusters

The computation complexity of SVDC is *O*(*n*_*j*_^2^ *r*_*j*_^3^), where *n*_*j*_ is the maximum number of documents in *A* ^(*i*)^  (1 ≤ *i* ≤ *k*) and *r*_*j*_ is the corresponding rank-*j* to approximate cluster submatrix *A* ^(*i*)^. Because the original term-document matrix *A* is partitioned into *k* cluster submatrices by clustering algorithm, we can estimate *n*_*j*_ ≈ *n*/*k* and *r*_*j*_ ≈ *r*/*k*. That is to say, the computation complexity of SVD compared to that of SVDC has been decreased by approximate *k* ^5^. The larger the value of *k* is, that is, the more the document clusters setting for a document collection is, the more computation complexity which will be saved by SVD on clusters in matrix factorization is. Although one may argue that clustering process in SVD on clusters will bring about computation complexity, in fact, the cost of clustering computation is far smaller than that of SVD. For instance, the computation complexity of *k*-Means clustering is *O*(*nkt*) [24], where *n* and *k* have the same meaning as those in SVD on clusters and *t* is the number of iterations. The computation complexity of clustering is not comparable to the complexity *O*(*n* ^5^) involved in SVD. The computation complexity of SOMs clustering is in the similar case with *k*-Means clustering.

### 3.5. Updating of SVD on Clusters

In rapidly changing environments such as the World Wide Web, the document collection is frequently updated with new documents and terms constantly being added, and there is a need to find the latent-concept subspace for the updated document collection. In order to avoid recomputing the matrix decomposition, there are two kinds of updates for an established latent subspace of LSI: folding in new documents and folding in new terms.

#### 3.5.1. Folding in New Documents

Let *D* denote *p* new document vectors to be appended into the original term-document matrix *A*; then *D* is *m* × *p* matrix. Thus, the new term-document matrix is *B* = (*A*, *D*). Then *B* = (*U*Σ*V* ^*T*^, *D*) = *U*Σ(*V* ^*T*^, Σ^−1^ *U* ^*T*^ *D*) = *U*Σ(*V*/*D* ^*T*^ *U*Σ^−1^)^*T*^. That is, if *D* is appended into the original matrix *A*, *V*_new_ = (*V*, *D* ^*T*^ *U*Σ^−1^) and *B* = *U*Σ*V*_new_^*T*^. However, here *V*_new_ is not an orthogonal matrix like *V*. So *B*_*k*_ is not the closest rank-*k* approximation matrix to *B* in terms of Frobenius norm. This is the reason why more documents are appended in *A*; more deteriorating effects are produced on the representation of the SVD approximation matrix using folding in method.

Despite this, to fold in *p* new document vectors *D* into an existing SVD decomposition, a projection $\bar{D}$ of *D* onto the span of the current term vectors (columns of *V*_*k*_^*T*^) is computed by (5). Here, *k* is the rank of the approximation matrix:

(5) $$\begin{array}{c} \bar{D}={\left(\;D^{T}U\Sigma^{-1}\;\right)}_{:,1:k}. \end{array}$$

As for folding in these *p* new document vectors *D* into the established SVDC decomposition of matrix *A*, we should decide firstly the cluster submatrices of *A* into which each vector in *D* should be appended. Next, using (5), we can fold in the new document vector into the cluster submatrix. Assuming that *d* is a new document vector of *D*, first, the Euclidean distance between *d* and *c*_*i*_ (*c*_*i*_ is the cluster center of cluster submatrix *A* ^(*i*)^) is calculated using (6), where *m* is the dimension of *d*, that is, the number of terms used in *A*. One has

(6) d−ci2=d1−ci,12+d2−ci,22+⋯+dm−ci,m2.

Second, *d* is appended into the *s*th cluster where *d* has the minimum Euclidean distance with *s*th cluster. That is,

(7) $$\begin{array}{c} s=\underset{i}{\arg\;}\underset{1\leq i\leq k}{\min}{\left\|\;d-c_{i}\;\right\|}^{2}. \end{array}$$

Third, (5) is used to update the SVD of *A* ^(*s*)^. That is,

(8) $$\begin{array}{c} \bar{d}={\left(\;d^{T}U\Sigma^{-1}\;\right)}_{:,1:r_{s}}. \end{array}$$

Here, *r*_*s*_ is the rank of approximation matrix of *A* ^(*s*)^. Finally, $\bar{A}$ is updated as $\bar{A}=(A_{r_{1}}^{\left(1\right)},\ldots ,A_{r_{s}}^{\left(s\right)},\ldots ,A_{r_{k}}^{\left(k\right)})$ with

(9) $$\begin{array}{c} A_{r_{s}}^{\left(s\right)}=U_{:,1:r_{s}}^{\left(s\right)}\Sigma_{1:r_{s},1:r_{s}}^{\left(s\right)}\left(\;V_{:,1:r_{s}}^{\left(s\right)}\;|\;{\bar{d}}^{T}\;\right). \end{array}$$

Thus, we finish the process of folding in a new document vector into SVDC decomposition and the centroid of *s*th cluster is updated with new document. The computational complexity of updating SVDC depends on the size of *U* and Σ because it involves only one-way matrix multiplication.

#### 3.5.2. Folding in New Terms

Let *T* denote a collection of *q* term vectors for SVD update. Then *T* is *q* × *n* matrix. Thus, we have the new term-document *C*, with *C* = (*A*/*T*) = (*A* ^*T*^, *T* ^*T*^)^*T*^. Then *C* = ((*U*Σ*V* ^*T*^)^*T*^, *T* ^*T*^)^*T*^ = (*U*Σ*V* ^*T*^/*T*) = (*U*/*TV*Σ^−1^)Σ*V* ^*T*^. That is, *U*_new_ = (*U*/*TV*Σ^−1^) and *C* = *U*_new_Σ*V* ^*T*^. Here, *U*_new_ is not an orthonormal matrix. So *C*_*k*_ is not the closest rank-*k* approximation matrix to *C* in terms of Frobenius norm. Thus, the more the terms being appended into the approximation matrix *A*_*k*_ are, the more the deviation between *A*_*k*_ and *A* which will be induced in document representation is.

Although the method specified above has a disadvantage of SVD for folding in new terms, we do not have better method to tackle this problem until now if no recomputing of SVD is desired. To fold in *q* term vectors *T* into an existing SVD decomposition, a projection, $\bar{T}$, of *T* onto the span of current document vectors (rows of *U*_*k*_) is determined by

(10) $$\begin{array}{c} \bar{T}={\left(\;TV_{k}\Sigma_{k}^{-1}\;\right)}_{1,1:k}. \end{array}$$

Concerning folding in an element *t* of *T*, the updating process of SVDC is more complex than that of SVD. First, the weight of *t* in each document of each cluster is calculated as

(11) $$\begin{array}{c} t^{\left(i\right)}=\left(\;w_{1}^{\left(i\right)},\ldots ,w_{j}^{\left(i\right)},\ldots ,w_{m_{i}}^{\left(i\right)}\;\right)\;\left(1\leq i\leq k\right). \end{array}$$

Here, *w*_*j*_^(*i*)^ is the weight of the new term *t* in the* j*th document of* i*th cluster submatrix *A* ^(*i*)^. *m*_*i*_ is the number of documents in *A* ^(*i*)^ and *k* is the number of clusters in the original term-document matrix *A*. Second, for each *A* ^(*i*)^  (1 ≤ *i* ≤ *k*) in $\bar{A}$ of Definition 2, the process of folding in a new term in SVD is used to update each *A* ^(*i*)^ shown in

(12) $$\begin{array}{c} {\bar{t}}^{\left(i\right)}={\left(\;t^{\left(i\right)}V^{\left(i\right)}\Sigma^{\left(i\right)-1}\;\right)}_{1,1:r_{i}}. \end{array}$$

Then, each *A*_*r*_*i*__^(*i*)^ is updated using

(13) $$\begin{array}{c} A_{r_{i}}^{\left(i\right)}=\left(\;\frac{U_{1:m_{i},1:r_{i}}^{\left(i\right)}}{{\bar{t}}^{\left(i\right)}}\;\right)\Sigma_{1:r_{i},1:r_{i}}^{\left(i\right)}V_{1:n,1:r_{i}}^{\left(i\right)T}. \end{array}$$

Finally, approximation term-document $\bar{A}$ of Definition 2 is reconstructed with all updated *A*_*r*_*i*__^(*i*)^ as

(14) $$\begin{array}{c} \bar{A}=\left[\;A_{r_{1}}^{\left(1\right)},\ldots ,A_{r_{i}}^{\left(i\right)},\ldots ,A_{r_{k}}^{\left(k\right)}\;\right]. \end{array}$$

Thus, we finish the process of folding *t* into SVDC decomposition. For folding *q* term vectors *T* into an existing SVDC decomposition, we need to repeat the processes of (11)–(14) for each element of *T* one by one.

## 4. Experiments and Evaluation

### 4.1. The Corpus

Reuters-21578 distribution 1.0 is used for performance evaluation as the English corpus and it is available online (http://www.daviddlewis.com/resources/testcollections/reuters21578/). It collects 21,578 news from Reuters newswire in 1987. Here, the documents from 4 categories as “crude” (520 documents), “agriculture” (574 documents), “trade” (514 documents), and “interest” (424 documents) are assigned as the target English document collection. That is, 2,042 documents from this corpus are selected for evaluation. After stop-word (we obtain the stop-words from USPTO (United States Patent and Trademark Office) patent full-text and image database at http://patft.uspto.gov/netahtml/PTO/help/stopword.htm. It includes about 100 usual words. The part of speech of English word is determined by QTAG which is a probabilistic parts-of-speech tagger and can be downloaded freely online: http://www.english.bham.ac.uk/staff/omason/software/qtag.html) elimination and stemming processing (Porter stemming algorithm is used for English stemming processing which can be downloaded freely online: http://tartarus.org/~martin/PorterStemmer/), a total amount of 50,837 sentences and 281,111 individual words in these documents is estimated.

TanCorpV1.0 is used as the Chinese corpus in this research which is available in the internet (http://www.cnblogs.com/tristanrobert/archive/2012/02/16/2354973.html). Here, documents from 4 categories as “agriculture,” “history,” “politics,” and “economy” are assigned as target Chinese corpus. For each category, 300 documents were selected randomly from original corpus, obtaining a corpus of 1,200 documents. After morphological analysis (because Chinese is character based, we conducted the morphological analysis using the ICTCLAS tool. It is a Chinese Lexical Analysis System. Online: http://ictclas.nlpir.org/), a total amount of 219,115 sentences and 5,468,301 individual words is estimated.

### 4.2. Evaluation Method

We use similarity measure as the method for performance evaluation. The basic assumption behind similarity measure is that document similarity should be higher for any document pair relevant to the same topic (intratopic pair) than for any pair relevant to different topics (cross-topic pair). This assumption is based on consideration of how the documents would be used by applications. For instance, in text clustering by *k*-Means, clusters are constructed by collecting document pairs having the greatest similarity at each updating.

In this research, documents in same category are regarded as having same topic and documents in different category are regarded as cross-topic pairs. Firstly, document pairs are produced by coupling each document vector in a predefined category and another document vector in the whole corpus, iteratively. Secondly, cosine similarity is computed for each document pair, and all the document pairs are sorted in a descending order by their similarities. Finally, (15) and (16) are used to compute the average precision of similarity measure. More details concerning similarity measure can be found in [9]. One has

(15) precisionpk=#  of  intra  -  topic  pairs  pj  where  j≤kk,

(16) average_precision=∑i=1mpim.

Here, *p*_*j*_ denotes the document pair that has the *j*th greatest similarity value of all document pairs. *k* is varied from 1 to *m* and *m* is the number of total document pairs. The larger the average precision (*p*_*k*_) is, the more the document pairs in same categories which are regarded as having same topic are. That is, the better performance is produced. A simplified method may be that *k* is predefined as fixed numbers such as 10, 20, and 200 (as suggested by one of the reviewers). Thus, (16) is not necessary. However, due to the lack of knowledge of the optimal *k*, we conjecture that an average precision on all possible *k* is more convincing for performance evaluation.

### 4.3. Experimental Results of Indexing

For both Chinese and English corpus, we carried out experiments for measuring similarities of documents in each category. When using SVDC in Algorithm 3 for LSI, the predefined number of clusters in *k*-Means clustering algorithm is set as 4 for both Chinese and English documents, which is equal to the number of categories used in both corpora. In SOMs clustering when using SVDC in Algorithm 4 for LSI, 10 × 10 array of neurons is set to map the original document vectors to this target space, and the limit on time iteration is set as 10,000. As a result, Chinese documents are mapped to 11 clusters and English documents are mapped to 16 clusters.  Table 2 shows the *F*-measure values [26] of the clustering results produced by *k*-Means and SOMs clustering, respectively. The larger the *F*-measure value, the better the clustering result. Here, *k*-Means has produced better clustering results than SOMs clustering algorithm.

Average precision (see (16)) on the 4 categories of both English and Chinese documents is used as the performance measure. Tables 3 and 4 are the experimental results of similarity measure on the English and Chinese documents, respectively. For SVD, SVDC, and ADE, the only required parameter to compute the latent subspace is preservation rate, which is equal to *k*/rank(*A*), where *k* is the rank of the approximation matrix. For IRR and SVR, besides the preservation rate, they also need another parameter as rescaling factor to compute the latent subspace.

To compare document indexing methods at different parameter settings, preservation rate is varied from 0.1 to 1.0 in increments of 0.1 for SVD, SVDC, SVR, and ADE. For SVR, its rescaling factor is set to 1.35, as suggested in [10] for optimal average results in information retrieval. For IRR, its preservation rate is set as 0.1 and its rescaling factor is varied from 1 to 10, the same as in [13]. Note that in Tables 3 and 4 for IRR, the preservation rate of 1 corresponds to rescaling factor 10, 0.9 to 9, and so forth. The baseline of TF*∗*IDF method can be regarded as pure SVD at preservation rate 1.0.

We can see from Tables 3 and 4 that for both English and Chinese similarity measure, SVDC with *k*-Means, SVDC with SOMs clustering, and SVD outperform other SVD based methods. In most cases, SVDC with *k*-Means and SVDC with SOMs clustering have better performances than SVD. This outcome validates our motivation of SVD on clusters in Section 3.1 that all documents in a corpus are not necessarily to be in a same latent space but in some different latent subspaces. Thus, SVD on clusters, which constructs latent subspaces on document clusters, can characterize document similarity more accurately and appropriately than other SVD based methods. Here, we regard that the variances of the mentioned methods are comparable to each other because they have similar values.

Considering the variances of average precisions on different categories, we admit that SVDC may not be a robust approach since its superiority is not obvious than SVD (as pointed out by one of the reviewers). However, we regard that the variances of the mentioned methods are comparable to each other because they have similar values.

Moreover, SVDC with *k*-Means outperforms SVDC with SOMs clustering. The better performance of SVDC with *k*-Means can be attributed to the better performance of *k*-Means than SOMs in clustering (see Table 2). When preservation rate declines from 1 to 0.1, the performances of SVDC with *k*-Means and SVD increase significantly. However, for SVDC with SOMs clustering, its performance decreases when preservation is smaller than 0.3. We hypothesize that SVDC with *k*-Means has effectively captured latent structure of documents but SVDC with SOMs clustering has not captured the appropriate latent structure due to its poor capacity in document clustering.

To better illustrate the effectiveness of each method, the classic *t*-test is employed [27, 28]. Tables 5 and 6 demonstrate the results of *t*-test of the performances of the examined methods on English and Chinese documents, respectively. The following codification of *P* value in ranges was used: “≫” (“≪”) means that *P* value is lesser than or equal to 0.01, indicating a strong evidence that a method produces a significant better (worse) similarity measure than another one; “<” (“>”) means that *P* value is larger than 0.01 and minor or equal to 0.05, indicating a weak evidence that a method produces a significant better (worse) similarity measure than another one; “~” means that *P* value is greater than 0.05, indicating that the compared methods do not have significant differences in performances. We can see that SVDC with *k*-Means outperforms both SVDC with SOMs clustering and pure SVD in both English and Chinese corpus. Meanwhile, SVDC with SOMs clustering has a very similar performance with pure SVD.

### 4.4. Experimental Results of Updating

**Figure 1:**
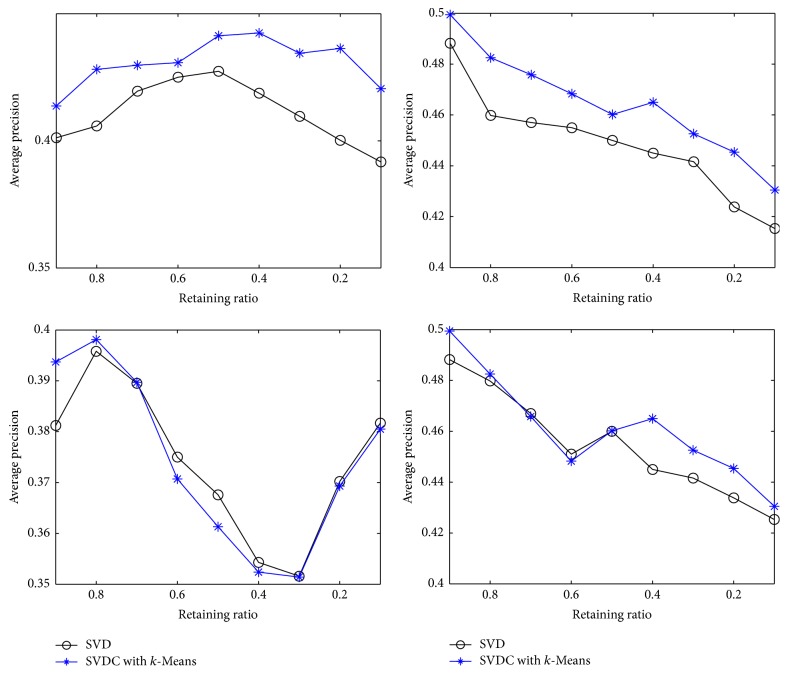
Similarity measure of SVDC with *k*-Means and SVD for updating; the preservation rates of their approximation matrices are set as 0.8.

Figure 1 is the performances of updating process of SVD on clusters in comparison with SVD updating. The vertical axis indicates average precision, and the horizontal axis indicates the retaining ratio of original documents for initial SVDC or SVD approximation. For example, the retaining ratio 0.8 indicates that 80 percentage of documents (terms) in the corpus are used for approximation and the left 20 percentage of documents (terms) are used for updating the approximation matrix. Here, the preservation rates of approximation matrices are set as 0.8 uniformly. We only compared SVDC with *k*-Means and SVD in updating because SVDC with SOMs clustering has not produced a competitive performance in similarity measure.

We can see from Figure 1 that, in folding in new documents, the updating process of SVDC with *k*-Means is superior to SVD updating on similarity measure. An obvious trend on their performance difference is that the superiority of SVDC with *k*-Means becomes more and more significant than SVD when the number of training documents declines. We conjecture that less diversity in latent spaces of small number of training documents can improve the document similarity in the same category.

In folding in new terms, SVDC with *k*-Means is superior to SVD as well. However, their performances drop dramatically in initial phase and increase after a critical value. This phenomenon can be explained as that when retaining ratio is large, the removal of more and more index terms from term-document matrix will hurt the latent structure of document space. However, when retaining ratio attains to a small value (the critical value), the latent structure of document space is decided principally by the appended terms which have larger number than remaining terms. For this reason, document similarities in the corpus are determined by the appended index terms. Furthermore, we observe that the critical value on Chinese corpus is larger than that on English corpus. This can be explained as that the number of Chinese index terms (21475) is much larger than that of English index terms (3269) but the number of Chinese documents (1200) is smaller than that of English documents (2402). Thus, the structure of Chinese latent space is much more robust than that of English latent space which is very sensitive to the number of index terms.

## 5. Concluding Remarks

This paper proposes SVD on clusters as a new indexing method for Latent Semantic Indexing. Based on the review on current trend of linear algebraic methods for LSI, we claim that the state of art of LSI roughly follows two disciplines: SVD based LSI methods and non-SVD based LSI methods. Then, with the specification of its motivation, SVD on clusters is proposed. We describe the algorithm of SVD on clusters with two different clustering algorithms: *k*-Means and SOMs clustering. The computation complexity of SVD on clusters, its theoretical analysis, and its updating process for folding in new documents and terms are presented in this paper. SVD on clusters is different from existing SVD based LSI methods in the way of eliminating noise from the term-document matrix. It neither changes the weights of singular values in Σ as done in SVR and ADE nor revises directions of singular vectors as done in IRR. It adapts the structure of the original term-document matrix based on document clusters. Finally, two document collections as a Chinese and an English corpus are used to evaluate the proposed methods using similarity measure in comparison with other SVD based LSI methods. Experimental results demonstrate that in most cases SVD on clusters outperforms other SVD based LSI methods. Moreover, the performances of clustering techniques used in SVD on clusters play an important role on its performances.

The possible applications of SVD on clusters may be automatic categorization of large amount of Web documents where LSI is an alternative for document indexing but with huge computation complexity and the refinement of document clustering where interdocument similarity measure is decisive for its performance. We admit that this paper covers merely linear algebra methods for latent sematic indexing. In the future, we will compare SCD on clusters with the topic based methods for Latent Semantic Indexing on interdocument similarity measure, such as Probabilistic Latent Semantic Indexing [29] and Latent Dirichlet Allocation [30].

## Figures and Tables

**Table 1:** Existing linear algebra methods for LSI.

Category	Abbreviation	Full name
SVD based decomposition for term-document matrix	IRR	Iterative Residual Rescaling
	SVR	Singular Value Rescaling
	ADE	Approximate Dimension Equalization

Non-SVD based decomposition for term-document matrix	SDD	Semidiscrete Decomposition
	LPI	Locality Preserving Indexing
	R-SVD	Riemannian-SVD

**Table 2:** *F*-measures of clustering results produced by *k*-Means and SOMs on Chinese and English documents.

Corpus	*k*-Means	SOMs clustering
Chinese	0.7367	0.6046
English	0.7697	0.6534

**Table 3:** Similarity measure on English documents of SVD on clusters and other SVD based LSI methods. PR is the abbreviation for “preservation rate” and the best performances (measured by average precision) are marked in bold type.

PR	SVD	SVDC (*k*-Means)	SVDC (SOMs)	SVR	ADE	IRR
1.0	**0.4373 **± **0.0236**	**0.4373 ± 0.0236**	**0.4373 ± 0.0236**	0.4202 ± 0.0156	0.3720 ± 0.0253	0.3927 ± 0.0378
0.9	0.4382 ± 0.0324	0.4394 ± 0.0065	**0.4400 ± 0.0266**	0.4202 ± 0.0197	0.2890 ± 0.0271	0.3929 ± 0.0207
0.8	0.4398 ± 0.0185	0.4425 ± 0.0119	**0.4452 ± 0.0438**	0.4202 ± 0.0168	0.3293 ± 0.0093	0.3927 ± 0.0621
0.7	0.4420 ± 0.0056	**0.4458 ± 0.0171**	0.4385 ± 0.0287	0.4089 ± 0.0334	0.3167 ± 0.0173	0.3928 ± 0.0274
0.6	0.4447 ± 0.0579	**0.4483 ± 0.0237**	0.4462 ± 0.0438	0.4201 ± 0.0132	0.3264 ± 0.0216	0.3942 ± 0.0243
0.5	0.4475 ± 0.0431	**0.4502 ± 0.0337**	0.4487 ± 0.0367	0.4203 ± 0.0369	0.3338 ± 0.0295	0.3946 ± 0.0279
0.4	0.4499 ± 0.0089	**0.4511 ± 0.0173**	0.4498 ± 0.0194	0.4209 ± 0.0234	0.3377 ± 0.0145	0.3951 ± 0.0325
0.3	0.4516 ± 0.0375	**0.4526 ± 0.0235**	0.4396 ± 0.0309	0.4222 ± 0.0205	0.3409 ± 0.0247	0.3970 ± 0.0214
0.2	0.4538 ± 0.0654	**0.4554 ± 0.0423**	0.4372 ± 0.0243	0.4227 ± 0.0311	0.3761 ± 0.0307	0.3990 ± 0.0261
0.1	0.4553 ± 0.0247	**0.4605 ± 0.0391**	0.4298 ± 0.0275	0.4229 ± 0.0308	0.4022 ± 0.0170	0.3956 ± 0.0185

**Table 4:** Similarity measure on Chinese documents of SVD on clusters and other SVD based LSI methods. PR is the abbreviation for “preservation rate” and the best performances (measured by average precision) are marked in bold type.

PR	SVD	SVDC (*k*-Means)	SVDC (SOMs)	SVR	ADE	IRR
1.0	**0.4312 ± 0.0213**	**0.4312 ± 0.0213**	**0.4312 ± 0.0213**	0.4272 ± 0.0200	0.3632 ± 0.0286	0.2730 ± 0.0168
0.9	0.4312 ± 0.0279	**0.4537 ± 0.0272**	0.4463 ± 0.0245	0.4272 ± 0.0186	0.3394 ± 0.0303	0.2735 ± 0.0238
0.8	0.4358 ± 0.0422	**0.4581 ± 0.0206**	0.4458 ± 0.0239	0.4273 ± 0.0209	0.3136 ± 0.0137	0.2735 ± 0.0109
0.7	0.4495 ± 0.0387	**0.4597 ± 0.0199**	0.4573 ± 0.0146	0.4273 ± 0.0128	0.3075 ± 0.0068	0.2732 ± 0.0127
0.6	0.4550 ± 0.0176	**0.4607 ± 0.0203**	0.4547 ± 0.0294	0.4273 ± 0.0305	0.3006 ± 0.0208	0.2730 ± 0.0134
0.5	0.4573 ± 0.0406	**0.4613 ± 0.0139**	0.4588 ± 0.0164	0.4273 ± 0.0379	0.2941 ± 0.0173	0.2729 ± 0.0141
0.4	0.4587 ± 0.0395	0.4624 ± 0.0098	**0.4659 ± 0.0255**	0.4275 ± 0.0294	0.2857 ± 0.0194	0.2726 ± 0.290
0.3	0.4596 ± 0.0197	**0.4644 ± 0.0183**	0.4582 ± 0.0203	0.4285 ± 0.0305	0.2727 ± 0.0200	0.2666 ± 0.242
0.2	0.4602 ± 0.0401	**0.4663 ± 0.0353**	0.4432 ± 0.0276	0.4305 ± 0.0190	0.2498 ± 0.0228	0.2672 ± 0.0166
0.1	0.4617 ± 0.0409	**0.4705 ± 0.0058**	0.4513 ± 0.0188	0.4343 ± 0.0193	0.3131 ± 0.0146	0.2557 ± 0.0188

**Table 5:** Results of *t*-test on the performances of similarity measure of SVD on clusters and other SVD based LSI methods in English corpus.

Method	SVDC with SOMs clustering	SVD
SVDC with *k*-Means	≫	≫
SVDC with SOMs clustering		>

**Table 6:** Results of *t*-test on the performances of similarity measure of SVD on clusters and other SVD based LSI methods in Chinese corpus.

Method	SVDC with SOMs clustering	SVD
SVDC with *k*-Means	>	>
SVDC with SOMs clustering		~
